# Clinical outcomes and temporal trends of immunological and non-immunological rare diseases in adult kidney transplant

**DOI:** 10.1186/s12882-021-02571-z

**Published:** 2021-11-17

**Authors:** Ester Gallo, Silvia Mingozzi, Alberto Mella, Fabrizio Fop, Roberto Presta, Manuel Burdese, Elena Boaglio, Maria Cristina Torazza, Roberta Giraudi, Gianluca Leonardi, Antonio Lavacca, Paolo Gontero, Omidreza Sedigh, Andrea Bosio, Aldo Verri, Caterina Dolla, Luigi Biancone

**Affiliations:** 1 Renal Transplant Center “A. Vercellone,” Nephrology, Dialysis, and Renal Transplant Division, “Città Della Salute e Della Scienza” Hospital, Department of Medical Sciences, University of Turin, Corso Bramante, 88-10126 Turin, Italy; 2 Division of Urology, Department of Surgical Sciences, Città Della Salute e Della Scienza Hospital and University of Turin, 10126 Turin, Italy; 3 Division of Vascular Surgery, Department of Thoracic-Vascular Surgery, Città Della Salute e Della Scienza Hospital, 10126 Turin, Italy

**Keywords:** Rare diseases, Kidney transplantation, Genetic renal diseases, Survival, Primary glomerulonephritis

## Abstract

**Background:**

Rare diseases (RDs) encompass many difficult-to-treat conditions with different characteristics often associated with end-stage renal disease (ESRD). However, data about transplant outcomes in adult patients are still lacking and limited to case reports/case series without differentiation between immunological/non-immunological RDs.

**Methods:**

Retrospective analysis among all adult kidney transplanted patients (KTs) with RDs (RDsKT group) performed in our high-volume transplantation center between 2005 and 2016. RDs were classified according to the Orphanet code system differentiating between immunological and non-immunological diseases, also comparing clinical outcomes and temporal trends to a control population without RDs (nRDsKT).

**Results:**

Among 1381 KTs, 350 patients (25.3%) were affected by RDs (RDsKTs). During a f/up > 5 years [median 7.9 years (4.8–11.1)], kidney function and graft/patient survival did not differ from nRDsKTs. Considering all post-transplant complications, RDsKTs (including, by definition, patients with primary glomerulopathy except on IgA nephropathy) have more recurrent and de-novo glomerulonephritis (14.6% vs. 9.6% in nRDsKTs; *p* = 0.05), similar rates of de-novo cancers, post-transplant diabetes, dysmetabolism, hematologic disorders, urologic/vascular problems, and lower infectious episodes than nRDsKTs (63.7% vs 72.7%; *p* = 0.013). Additional stratification for immunological and non-immunological RDsKTs or transplantation periods (before/after 2010) showed no differences or temporal trends between groups.

**Conclusions:**

Kidney transplant centers are deeply involved in RDs management. Despite their high-complex profile, both immunological and non-immunological RDsKTs experienced favorable patients’ and graft survival.

**Supplementary Information:**

The online version contains supplementary material available at 10.1186/s12882-021-02571-z.

## Background

A medical condition in Europe is classified as a rare disease (RDs) if its prevalence is less than one case per 2000 (0,05%) with an estimated incidence of two cases/100,000 inhabitants/year. According to this assumption, ∼5000–7000 RDs affect approximately 27 to 36 million people (6–8%) of the European population [1]. Most of these conditions are genetically inherited and affect patients for his/her entire lifetime [2, 3], posing both clinical and welfare issues (availability, affordability, and costs for diagnosis and treatment combined with adverse outcomes) [1, 4, 5].

All these considerations pave the way for international initiatives to improve the healthcare needs of patients with RDs. In this context, the European Commission strongly suggests adopting the Orphanet classification system (ORPHA code) to classify RDs [6]. The Orpanet database encompasses more than 6700 RDs [7], including, beyond a classification system, much information about causative genes and possible therapeutic strategies (i.e., with orphan drugs).

In the nephrological setting, most glomerulonephritis could be classified as RDs; at the same time, many RDs could directly determine end-stage renal disease (ESRD) or be a crucial part of the clinical picture of ESRD patients. To date, the overall prevalence of renal replacement therapy (RRT) in Europe is estimated at 1000 per million population [8], but data about RDs in this population are scarce and outdated [9, 10]. In the most recent paper by Wuhl et al. [3], derived from the ERA-EDTA Registry analysis, 12.4% of all RRT patients in the Euro area would be affected by RDs. Interestingly, although RDs have been frequently identified during the pediatric lifetime, only 5.8% of RRT patients were younger than 20 years [3].

Not surprisingly, epidemiological or survival analyses about kidney transplantation (KT) in RDs are still lacking; most of the available studies described case reports/case series focusing on specific disease [11–14].

Our study aimed to (I) determine the prevalence of RDs in our population of adult KT recipients over time and (II) define whether and how the presence of RDs, also differentiating between immunological and non-immunological conditions, influences the KT outcomes comparing this subgroup to the overall population.

## Methods

### Study design and included population

We conducted a retrospective analysis including all consecutive adult KTs performed at the *Renal Transplant Center “A. Vercellone,” AOU Città Della Salute e Della Scienza Hospital, Turin,* in the period between 01/01/2005–31/12/2016. All pediatric KTs and patients without a determined cause of ESRD were excluded.

Based on the absence of a specific classification for RDs in RRT, we identified RDs in our population (RDsKT group) adopting the Orphanet database codification system (Table S1, Additional information). Therefore, all primary glomerulopathies were included except on IgA nephropathy (incidence rate 2.5/100000/year) [15]; patients without any histological assessment on native kidneys but with clinically documented hypertensive kidney disease and/or no features of immunological damage (i.e., no history of nephritic/nephrotic syndrome, hypocomplementemia, c- or p-ANCA, ANA, ENA, Ab anti-PLA_2_R or anti-GBM) were defined as “chronic glomerulopathy” and eventually included in the control group. CAKUT group merged patients with congenital renal dysplasia or hypoplasia with or without urinary tract malformation, chronic pyelonephritis due to congenital obstructive uropathy, or vesical-ureteric reflux without obstruction, and Prune-Belly syndrome. KTs with ESRD due to primary/secondary amyloidosis and the familial Mediterranean fever were all encompassed in the amyloidosis group.

**Fig. 1:**
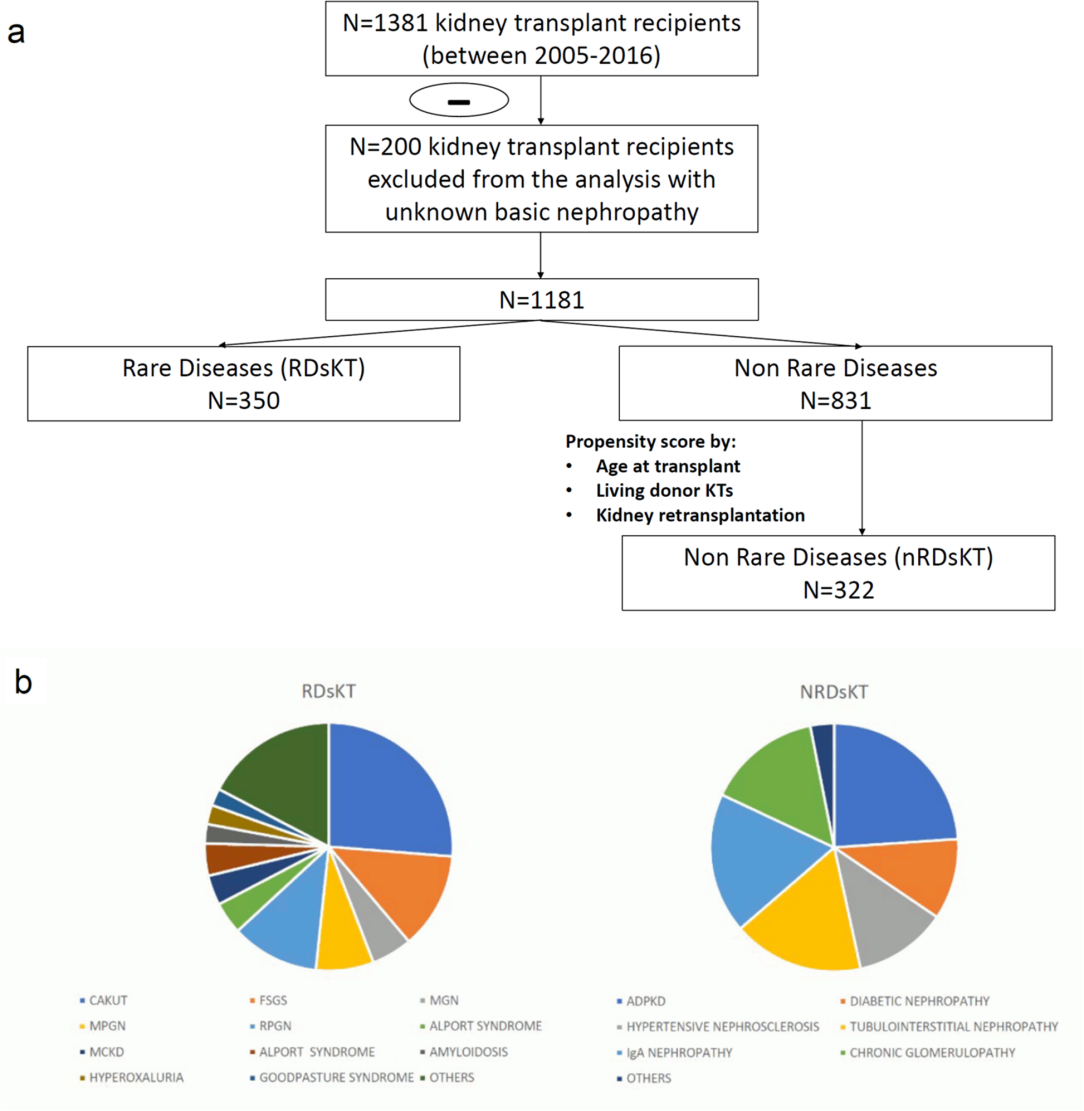
Flow chart and graphical schematization of (**a**) selection criteria for the studied population (**b**) disease classification in both groups. RDsKT: rare diseases kidney transplant. nRDsKT: non-rare diseases kidney transplant. CAKUT: congenital anomalies of the kidneys and the urinary tract. FSGS: focal segmental glomerulosclerosis MGN: membranous glomerulonephritis. MPGN: membranoproliferative glomerulonephritis. RPGN: rapidly progressive glomerulonephritis. MCKD: medullary cystic kidney disease. ADPKD: autosomal dominant polycystic kidney disease

To appropriately analyze this population’s clinical characteristics and functional outcomes, we compared RDsKT to a control group of KTs without RDs (nRDsKT group) matched through a propensity score model (ratio 1:1; the decade of age at KT, number of living-donor, and previous kidney transplants as dependent variables). Figure 1a illustrates the selection process’s flowchart.

We retrospectively analyzed the demographic and clinical characteristics of each group. All patient data were registered in our database and collected until 01/05/2019.

Evaluation of renal functional outcomes included serum creatinine (sCr) and proteinuria (uPt) at discharge and 1, 2, and 5 years after KT. Information about immunosuppressive therapy (also including induction protocol) was also collected.

Data about allograft biopsies, primarily performed for cause (i.e., significant or unexplained increase of sCr > 25% from baseline, proteinuria, or both) were analyzed and reviewed according to the 2017 Banff classification [16]. Post-transplant glomerulonephritis cases were stratified as recurrent/de novo or undetermined cause based on available pre-transplant histological data.

For both groups, post-transplant complications (infectious episodes, de-novo cancers, post-transplant diabetes, dysmetabolism, hematologic disorders, urologic/vascular problems), and patient/graft survival (including the cause of patients’ death or allograft failure) were investigated.

The study was performed in adherence to the last version of the Helsinki Declaration and the Principles of the Declaration of Istanbul on Organ Trafficking and Transplant Tourism. All KT recipients signed informed consent, including their permission to have data from their medical records used in research. All the methods are approved by the institutional Ethical Committee (*Comitato Etico Interaziendale A.O.U. Città Della Salute e Della Scienza di Torino - A.O. Ordine Mauriziano - A.S.L. Città di Torino*) approval, resolution number 1449/2019 on 11/08/2019 (“TGT observational study”).

### Statistical analysis

Statistical analysis was performed with SPSS (IBM SPSS Statistics, vers. 25.0.0). Continuous variables are presented, according to their distribution, as mean ± SD or as median (min-max). Inter-group differences were analyzed with t-test or Mann-Whitney test, respectively.

We expressed categorical variables as fractions, and Pearson’s χ2 or, for small samples, Fisher’s exact test was adopted to compare groups. The odds ratios (OR) with 95% CI were used as a measure of relative risk.

Survival analysis was performed with the Kaplan-Meier method, comparing groups with the Log Rank test. The significance level (α) was set at *p* < 0.05 for all tests.

## Results

### Prevalence of RDs and characteristics of the studied population

During the studied period, 350/1381 KTs (25.3%) were performed in patients with RDs. The leading causes of ESRD in RDsKTs were CAKUT syndrome, focal segmental glomerulosclerosis, and rapidly progressive glomerulonephritis (Fig. 1b).

Table 1 shows baseline characteristics in RDsKTs and nRDsKTs, including the immunosuppressive regimen at transplantation (data stratified for RDs are available in Table S2, Additional information). No significant demographic difference was observed between groups, except for the higher number of combined KTs in the nRDsKT group, primarily determined by diabetic nephropathy and adult polycystic kidney disease (ADPKD) patients necessitating pancreas/kidney and liver/kidney transplant, respectively.

**Table 1:** Demographic characteristics of the studied population at kidney transplant

	RDsKT ***n*** = 350	nRDsKT ***n*** = 322	***p***
**Recipient characteristics**			
**Age at transplant (yrs), mean (SD)**	50.0 (38.0–59.0)	51.0 (42.0–61.0)	0.064
**Gender male, N. (%)**	208 (59.4)	202 (62.7)	0.380
**Previous transplantation, N. (%)**	68 (19.4)	54 (16.8)	0.106
**Combined transplantation, N. (%)**			**0.005**
**+ heart**	1 (0.3)	0 (0)	
**+ liver**	8 (2.29)	10 (3.11)	
**+ pancreas**	0 (0)	13 (4.04)	
**Time since dialysis (yrs), mean (SD)**	3.0 (1.6–5.6)	3.1 (1.8–5.6)	0.815
**Donor characteristics**			
**Age (yrs), mean (SD)**	55.0 (45.0–65.0)	57.0 (48.0–67.0)	0.052
**Gender male, N. (%)**	180 (51.4)	161 (50)	0.711
**Deceased donor, N. (%)**	318 (90.9)	300 (93.2)	0.271
**Dual kidney transplantation, N. (%)**	10 (2.9)	4 (1.2)	0.143
**Delayed Graft Function**^a^**, N. (%)**	76 (22.6)	73 (23.2)	0.866
**Immunology at the time of transplantation**			
**HLA mismatches, median (IQR)**	3 (2–4)	3 (2–4)	0.214
**vPRA (%), median (IQR)**			
**Class I**	13.6 (0.0–37.3)	9.2 (0.0–51.1)	0.624
**Class II**	1.88 (0.0–46.1)	0.5 (0.0–51.6)	0.851
**Total**	27.1 (4.8–72.4)	35.7 (4.8–72.4)	0.716
**Time between the first evaluation at pre-transplant unit and the active waiting-list admittance (months)**^b^**, median (IQR)**	6.0	4.3 (2.3–8.2)	**0.038**
**Time on active waiting list (months), median (IQR)**	9.5 (3.0–26.1)	8.6 (3.1–28.0)	0.886
**Length of stay (days)**^c^**, median (IQR)**	17 (13–26)	18 (14–26.75)	0.720
**Induction therapy**			0.440
**No induction, N. (%)**	2 (0.6)	1 (0.3)	
**Anti-CD25 therapy, N. (%)**	308 (88.0)	291 (90.4)	
**Thymoglobuline, N. (%)**	16 (4.6)	12 (3.7)	
**Anti-CD25 therapy + Thymoglobuline, N. (%)**	16 (4.6)	8 (2.5)	
**At discharge**^d^			
**Immunosuppressive therapy**			0.840
**Tacrolimus, N. (%)**	299 (85.4)	286 (88.8)	
**plus mycophenolate mofetil/azathioprine and steroids, N. (%)**	242 (72)	225 (71.2)	
**plus mycophenolate mofetil/azathioprine or steroids, N. (%)**	48 (14.3)	48 (15.2)	
**Cyclosporine A, N. (%)**	29 (8.3)	20 (6.2)	
**plus mycophenolate mofetil/azathioprine and steroids, N. (%)**	19 (5.7)	14 (4.4)	
**No Calcineurin inhibitor, N. (%)**	8 (2.3)	10 (3.1)	
**mTor inhibitor, N. (%)**	6 (1.7)	28 (8.7)	

*vPRA* Virtual panel reactive antibody, *sCr* Serum creatinine

^a^Intended as use of dialysis in the first week after kidney transplantation

^b^Among 211 RDsKTs and 188 nRDsKTs transplanted from 2010 onwards, for whom data were available (109 and 82, respectively)

^c^Among 211 RDsKTs and 188 nRDsKTs transplanted from 2010 onwards, for whom data were available

^d^Among the 336 RDsKTs and 316 nRDsKTs with functioning kidney graft at discharge

The mean recipient age and M/F ratio were similar among groups (48.8 ± 13.4 years in RDsKTs vs. 50.7 ± 13.0 in nRDsKTs, and 59.4% males vs. 62.7%, respectively). Interestingly, RDsKTs experienced a long time between the first evaluation at our pre-transplant unit and the final admission on the active waiting list [6.0 months (3.5–10.1) vs. 4.3 (2.3–8.2); *p* = 0.038].

Kidney function and immunosuppressive regimens at discharge and 1, 2, and 5 years post-KT are summarized in Table 2. Median sCr at discharge was slightly better in RDsKTs (1.58 mg/dL vs. 1.72 mg/dL in RDsKT group; *p* = 0.026); during the f/up time [7.9 years (4.8–11.1) in RDsKTs vs. 8.5 (5.3–11.5) in nRDsKTs] no significant differences in kidney function tests were observed at any time-point. No difference in maintenance immunosuppressive medications was even documented, also considering Mycophenolate Mofetil/Azathioprine and Steroids.

**Table 2:** Kidney function and maintenance immunosuppressive therapy in studied population during the follow-up

	RDsKT (***n*** = 350)	nRDsKT(***n*** = 322)	***P***
**At discharge**			
**sCr (mg/dL), median (IQR)**	1.58 (1.27–2.00)	1.72 (1.30–2.10)	**0.026**
**Proteinuria (g/day), median (IQR)**	0.3 (0.2–0.5)	(0.2–0.5)	0.581
**At 1 year**^a^			
**sCr (mg/dL), median (IQR)**	1.48 (1.20–1.86)	1.58 (1.20–2.00)	0.064
**Proteinuria (g/day), median (IQR)**	0.19 (0.12–0.30)	0.19 (0.12–0.33)	0.587
**At 2 years**^b^			
**sCr (mg/dL), median (IQR)**	1.40 (1.15–1.90)	1.45 (1.19–1.80)	0.771
**Proteinuria (g/day), median (IQR)**	0.18 (0.12–0.33)	0.20 (0.12–0.34)	0.891
**At 5 years**^c^			
**sCr (mg/dL), median (IQR)**	1.40 (1.15–1.90)	1.44 (1.15–1.90)	0.826
**Proteinuria (g/day), median (IQR)**	0.18 (0.12–0.37)	0.20 (0.12–0.40)	0.337
**Immunosuppressive therapy**			0.209
**Tacrolimus, N. (%)**	174 (82.9)	178 (82.4)	
**plus mycophenolate mofetil/azathioprine and steroids, N. (%)**	52 [17]	41 (19.4)	
**plus mycophenolate mofetil/azathioprine or steroids, N. (%)**	76 (36.5)	77 (36.5)	
**Cyclosporine A, N. (%)**	17 (8.1)	18 (8.3)	
**plus mycophenolate mofetil/azathioprine and steroids, N. (%)**	5 (2.4)	5 (2.4)	
**plus mycophenolate mofetil/azathioprine or steroids, N. (%)**	8 (3.8)	8 (3.8)	
**No Calcineurin inhibitor, N. (%)**	17 (8.1)	14 (6.5)	
**mTor inhibitor, N. (%)**	46 (21.9)	48 (22.2)	

*sCr* Serum creatinine

^a^Among the 326 RDsKTs and 306 nRDsKTs with functioning kidney graft after one year from transplantation

^b^Among the 315 RDsKTs and 292 nRDsKTs with functioning kidney graft after two years from transplantation

^c^Among the 210 RDsKTs and 216 nRDsKTs with functioning kidney graft after five years from transplantation

### Analysis of post-transplant complications and patient/graft survival

Clinical complications after KT are reported in Table 3. As expected, considering the adopted classification system, RDsKT group has a higher rate of recurrent and de-novo glomerulonephritis (14.6% vs. 9.6%; *p* = 0.05), mainly determined by patients with membranous nephropathy and focal-segmental glomerulosclerosis (57.9 and 11.1% of recurrency, respectively) in RDs group and IgA nephropathy in nRDS group (71.4%). Infectious episodes occurred more frequently in nRDsKTs (*p* = 0.013), mainly due to increased viral events in this group. Moreover, biopsy-proven rejection episodes (also stratifying for T-Cell/antibody-mediated subtypes), neoplasia (including skin, solid, and hematolymphoid tumors), post-transplant diabetes, and hematologic complications occurred without differences between RDsKTs and nRDsKTs.

**Table 3:** Post-transplant complications and kidney transplant outcomes in the studied population

	RDsKT *n* = 350	nRDsKT *n* = 322	*P*
**Glomerulonephritis, N. (%)**	51 (14.6)	31 (9.6)	**0.050**
**Recurrent, N.**	30	14	
**De novo, N.**	11	10	
**Undetermined, N.**	10	7	
**Rejection (main histologic diagnosis)**^a^**, N. (%)**	66 (18.9)	62 (19.3)	0.900
**AMR, N.**	47	39	
**TCMR, N.**	19	23	
**Infection (≥ 1 episode), N. (%)**	223 (63.7)	234 (72.7)	**0.013**
**UTI, No. (%)**	130 (37.1)	133 (41.3)	0.269
**Recurrent UTI, N. (%)**	47 (13.4)	39 (12.1)	0.610
**Urosepsis, N. (%)**	27 (7.7)	36 (11.1)	0.124
**Viral infections, N. (%)**	96 (27.4)	114 (35.4)	**0.026**
**Tumors (≥ 1 episode), N. (%)**	52 (14.9)	58 (18.0)	0.270
**Skin tumors, N. (%)**	28 (8.0)	27 (8.4)	0.856
**Solid tumors, N. (%)**	24 (6.8)	31 (9.6)	0.191
**Hematolymphoid tumors, N. (%)**	1 (0.3)	4 (1.2)	0.149
**Post-transplant diabetes, N. (%)**	54 (15.4)	54 (16.8)	0.640
**Post-transplant dysmetabolism, N. (%)**	102 (29.1)	82 (25.5)	0.290
**Hematologic complications, N. (%)**	65 (18.6)	60 (18.6)	0.980
**Urologic complications, N. (%)**	54 (15.4)	50 (15.5)	0.970
**Vascular complications, N. (%)**	30 (8.6)	40 (12.4)	0.100
**Transplant failure, N. (%)**	58 (16.6)	57 (17.7)	0.700
**Chronic AMR, N.**	7	14	
**Acute rejection, N.**	5	6	
**Glomerulonephritis (recurrent), N.**	13	6	
**Glomerulonephritis (de novo), N.**	2	1	
**Interstitial fibrosis/tubular atrophy, N.**	4	9	
**Deceases with functioning kidney transplant, N. (%)**	23 (6.6)	28 (8.7)	0.300
**Cardiovascular, N.**	8	5	
**Infection/sepsis, N.**	6	14	
**Cancer, N.**	6	7	
**Other, N.**	3	2	

*AMR* Antibody-mediated rejection, *TCMR* T-cell-mediated rejection, *UTI* Urinary Tract Infections

^a^ Overall, rejection episodes observed in RDsKTs and nRDsKTs were 105 (76 [72.4%] AMR and 25 [23.8%] TCMR) and 90 (62 [68.9%] AMR and 26 [28.9%] TCMR), respectively

**Fig. 2:**
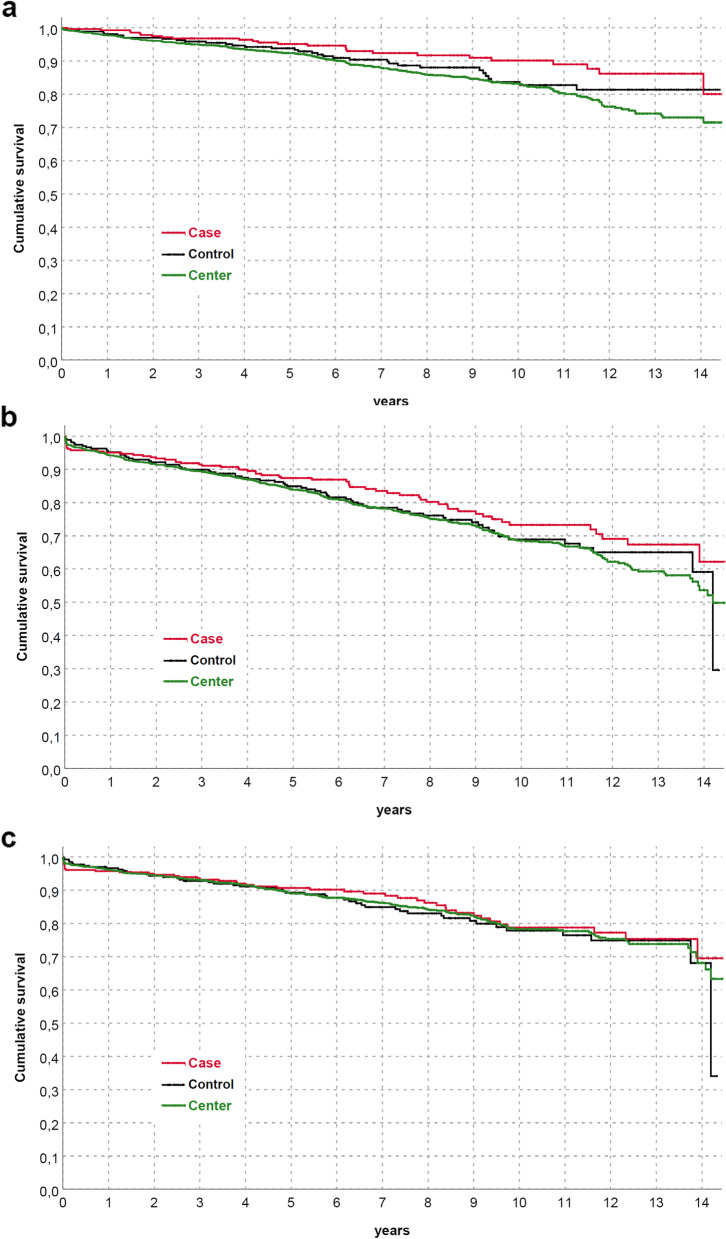
Kaplan-Meier curves for (**a**) all studied population, (**b**) overall graft survival (**c**) death-censored graft survival. Patient and kidney survivals showed no differences between RDsKTs and nRDsKT [*p* = 0.156 in (**a**); *p* = 0.245 in (**b**); *p* = 0.488 in (**c**)]

**Fig. 3:**
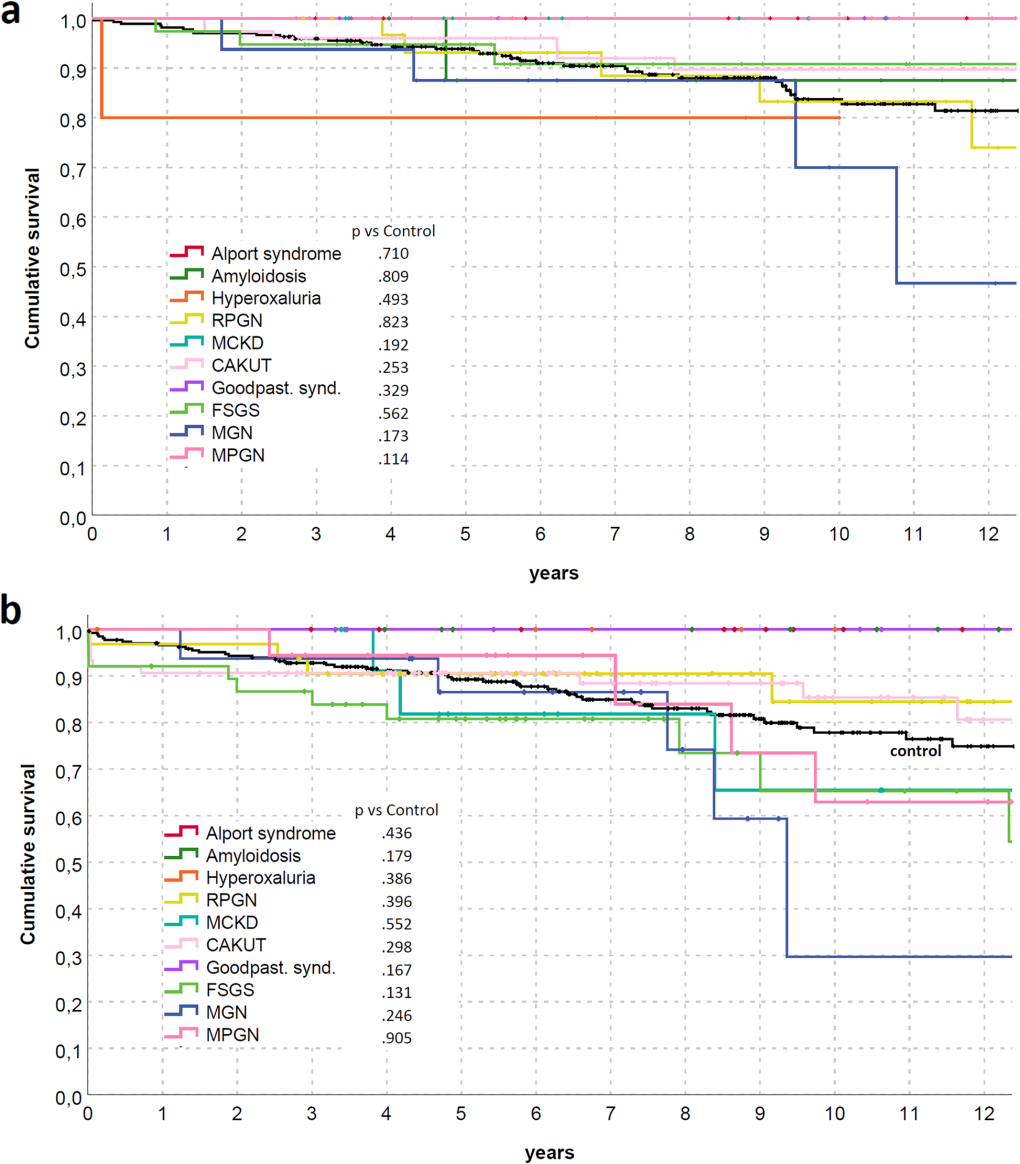
Kaplan-Meier curves according to RDs for (**a**) patients (**b**) graft (death-censored). Patient and kidney survivals did not differ according to the type of rare disease and compared with the nRDsKT group

**Fig. 4:**
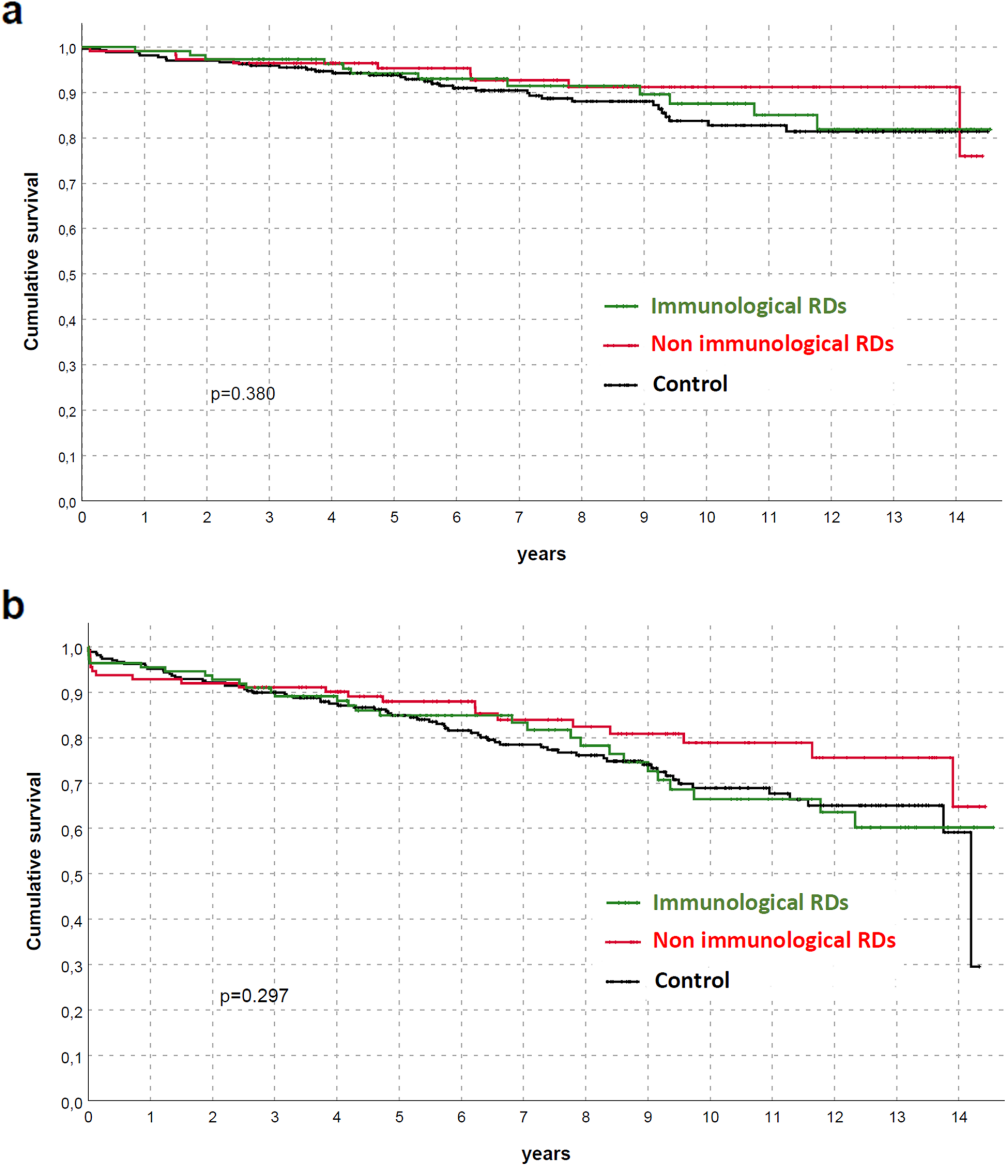
Kaplan-Meier curves according to immunological and non-immunological RDs for (**a**) patients (**b**) graft (death-censored). Patient and kidney survivals did not differ between immunological and non-immunological RDs and compared with the nRDsKT group

**Fig. 5:**
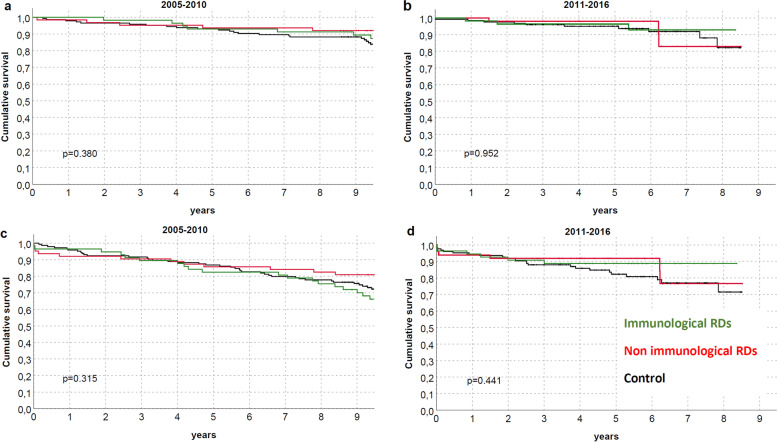
Kaplan-Meier curves according to immunological and non-immunological RDs for (**a**,**c**) patients (**b**,**d**) graft (death-censored) stratified for different time-points. Patient and kidney survivals did not differ between immunological, non-immunological RDs and nRDsKT group, also differentiating for KTs performed before (**a**, **c**) and after (**b**, **d**) the median f/up time

**Fig. 6:**
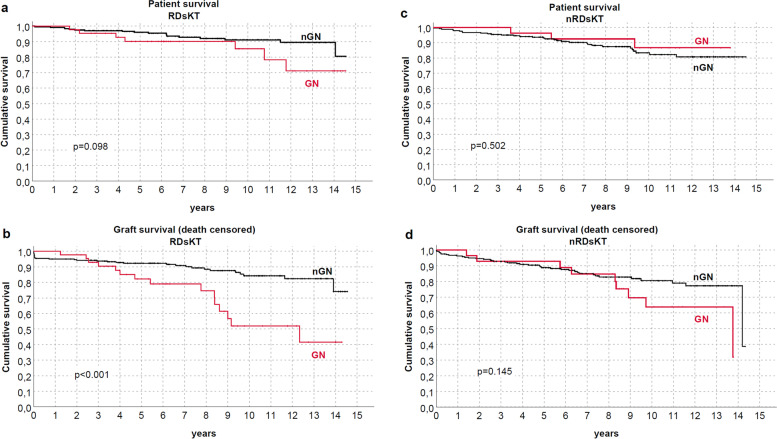
Kaplan-Meier curves according to recurrent/de novo glomerulonephritis for (**a**, **c**) patients (**b**,**d**) graft (death-censored). Negative graft survival was observed in patients with recurrent/de novo glomerulonephritis (GN) vs. nGN among the RDsKT group (*p* < 0.001)

**Fig. 7:**
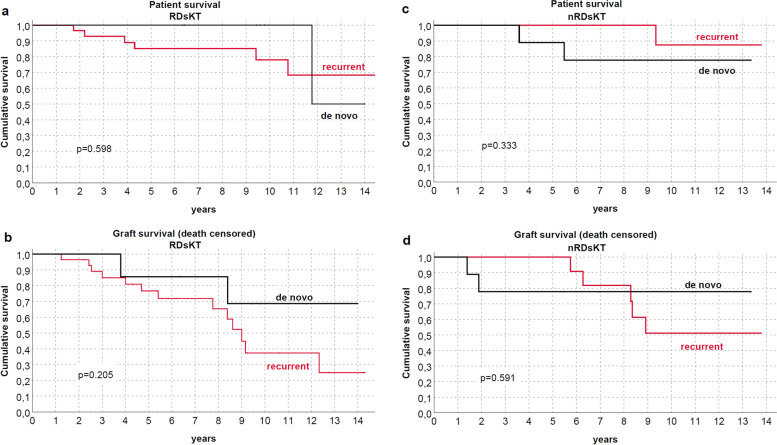
Kaplan-Meier curves in recurrent and de novo glomerulonephritis for (**a**, **c**) patients (**b**,**d**) graft (death-censored). Patient and kidney survivals did not differ between recurrent and de novo glomerulonephritis in RDsKT and nRDsKT groups

Both patients and graft survival were strictly similar between groups (Fig. 2), also differentiating for RDs (Fig. 3) or between immunological and non-immunological conditions (Fig. 4). Similar trends were also observed comparing patients who received KTs before and after the median f/up time (Fig. 5). As expected, patients with recurrent/de-novo glomerulonephritis have a reduced graft survival, with a significant difference in RDs group (Fig. 6). No difference was already noted stratifying between recurrent and de novo conditions (Fig. 7).

At least 58 patients in the RDsKT group and 57 nRDsKTs experienced transplant failure; the leading causes were recurrent glomerulonephritis and chronic antibody-mediated rejection. Death with functioning graft occurred with similar percentages in both groups (6.6% vs. 8.7%; *p* = 0.300). Deaths were mainly dependent on septic events, cardiovascular or neoplastic complications without differences between RDsKTs and nRDsKTs.

## Discussion

Identification, management, and treatment of RDs represent a challenging question for every health care system [5, 17, 18]. First of all, the heterogeneity of classification systems limited the generalizability of literature data. Furthermore, the difference in the availability of specific diagnostic testing (i.e., genetic analysis) reflects the high variability in RDs incidence, such as for Alport Syndrome [19].

All these considerations appear crucial in RRT patients considering that many ESRD causes could be categorizable as RDs. However, to the best of our knowledge, no study has to date specifically investigated the prevalence of RDs in adult KTs focusing on renal and patient outcomes and comparing them to the overall transplanted population.

Our analysis found that a significant number of KTs (25% of all consecutive transplanted patients between January 2005 and December 2016) have a documented RDs (classified according to ORPHA code) as ESRD cause or significant medical condition. The prevalence of RDs, similar to Wuhl et al. [3], dramatically differs from those reported in the general population [7, 20, 21] (Table 4).

**Table 4:** RDs prevalence in our kidney transplanted population compared to available data in the general population

Rare disease	Kidney transplant recipients^a^	General population [6, 19, 20]
**CAKUT**	6.8%	0.1–0.3%
**FSGS**	3.25%	Unknown
**MGN**	1.37%	Unknown
**MPGN**	1.95%	0.01–0.05%
**RPGN**	2.96%	Unknown
**MCKD**	1.01%	0.001–0.009%
**ALPORT SYNDROME**	1.08%	Unknown
**GOODPASTURE SYNDROME**	0.58%	0.0001–0.0009%
**HYPEROXALURIA**	0.65%	0.0001–0.0009%
**AMYLOIDOSIS**	0.65%	Unknown

*CAKUT* Congenital anomalies of the kidney and urinary tract, *FSGS* Focal segmental glomerulosclerosis, *MGN* Membranous glomerulonephritis, *MPGN* Membranoproliferative glomerulonephritis, *RPGN* Rapidly progressive glomerulonephritis, *MCKD* Medullar cystic kidney disease

^a^estimated on the studied population of consecutive adult KTs (*n* = 1381) performed at our center between January 2005 and December 2016

During the significant f/up time, RDsKTs experienced a favorable clinical course without differences in graft/patient survival and kidney function tests compared to nRDsKTs, even stratifying for immunological and non-immunological conditions. Recurrent and de novo glomerulonephritis were effectively diagnosed in both groups, with a prevalent incidence of FSGS and MGN in RDs and IgA nephropathy in nRDs. All these conditions have a detrimental impact on graft survival [22, 23] which was more evident in RDs due to the higher occurrence and the more pronounced effect of some diseases (i.e., FGSG, MGN, and MPGN) on short-time graft outcomes [24–26].

Post-transplant complications apart from the expected higher rate of recurrent glomerulonephritis also occur similarly in both groups. We noted the absence of UTI increase in nRDs patients, which included subjects with CAKUT syndrome (normally exposed to increase UTI risk) [27]. Exploring this finding, we identified specific attention in this subgroup to antibiotic prophylaxis (which was generally prolonged to up to 1 week) and to a rapidly ureteral catheter/double J ureteral stent removal to prevent colonization.

However, although patients have similar rates of complications, the pre-transplant balance of RDs patients was longer, reflecting the need for a specific and time-consuming multidisciplinary approach (i.e., for correct pre-transplant diagnosis, analysis of all comorbid conditions, evaluation of possible recurrence rate on KT, limitation in living donor transplantation for hereditary forms).

## Conclusions

Transplant units are at the crossroads of not-so-rare RDs, resulting in significant involvement in their challenging management. However, RDs patients who received KTs have favorable outcomes with comparable complications and graft failure rates to those observed in nRDsKTs patients. Our data also strengthen the importance of an accurate monitorization to prevent/identify recurrent glomerulonephritis in those conditions at high risk (i.e., FSGS, MGN, MPGN) and rapidly diagnose de novo cases.

Further analyses (for example, introducing a tailored Transplant Registry for RDs within the Italian and international transplant community and implementing electronic health records [28]) are highly needed to expand and confirm our positive results.

## Supplementary Information

**Additional file 1: Table S1.** Rare disease classification (according to Orpha-code) in the studied population. **Table S2.** Baseline characteristics and renal function tests among the most frequent RDs subgroups of the studied population.

## Data Availability

The datasets used and/or analysed during the current study are available from the corresponding author on reasonable request.

## Abbreviations

RDs: Rare diseases
ESRD: End-stage renal disease
KTs: Kidney transplanted patients
RDsKT: Kidney transplanted patients with rare diseases
nRDsKT: Kidney transplanted patients without rare diseases (control group)
RRT: Renal replacement therapy
ADPKD: Adult polycystic kidney disease (ADPKD)
