# Supplementary material for: Clinical outcomes and temporal trends of immunological and non-immunological rare diseases in adult kidney transplant
Source: BMC Nephrol. 2021 Nov 17;22:386. doi: 10.1186/s12882-021-02571-z (PMC8600810; doi:10.1186/s12882-021-02571-z)
Supplement: Supplementary file 1 — Additional file 1: Table S1. Rare disease classification (according to Orpha-code) in the studied population. Table S2. Baseline characteristics and renal function tests among the most frequent RDs subgroups of the studied population. [file 12882_2021_2571_MOESM1_ESM.docx]

**Table S1.** Rare disease classification (according to ORPHA code) in the studied population.

|  | n | ORPHA code |
| --- | --- | --- |
| **ALPORT Syndrome** | 15 | 63 |
| **AMYLOIDOSIS** | 9 | NA |
| Primary AMYLOIDOSIS | 6 | 69 |
| Secondary AMYLOIDOSIS | 3 | 85445 |
| **FAMILIAL MEDITERRANEAN FEVER** | 1 | 342 |
| **BARTTER Syndrome** | 2 | 112 |
| **CYSTINOSIS** | 1 | 213 |
| **CONNECTIVE TISSUE DISEASE** | 3 | NA |
| MIXED CONNECTIVE TISSUE DISEASE | 2 | 809 |
| UNDIFFERENTIATED CONNECTIVE TISSUE SYNDROME | 1 | 90002 |
| **CRYOGLOBULINEMIA** | 1 | 91139 |
| **NEPHROGENIC DIABETES INSIPIDUS** | 1 | 223 |
| **GALACTOSIALIDOSIS** | 1 | 351 |
| **PRIMARY HYPEROXALURIA** | 9 | 416 |
| **BOURNEVILLE Syndrome** | 1 | 805 |
| **BEHCET DISEASE** | 1 | 117 |
| **HEMOLYTIC UREMIC SYNDROME** | 6 | NA |
| HEMOLYTIC UREMIC SYNDROME ASSOCIATED WITH SHIGA TOXIN-PRODUCING ESCHERICHIA COLI | 2 | 90038 |
| ATYPICAL HEMOLYTIC UREMIC SYNDROME | 4 | 2134 |
| **VASCULITIS** | 41 | NA |
| **RCAD Syndrome** | 1 | 93111 |
| **MEDULLARY CYSTIC KIDNEY DISEASE** | 14 | 34149 |
| **SARCOIDOSIS** | 2 | 797 |
| **SCHONLEIN HENOCH PURPURA** | 3 | 761 |
| **SYSTEMIC SCLEROSIS** | 1 | 220393 |
| **LAURENCE-MOON Syndrome** | 3 | 2377 |
| **SENIOR-LOKEN Syndrome** | 2 | 3156 |
| **PRIMARY GLOMERULAR DISEASE** | 97 | NA |
| FSGS | 44 | 93218 |
| MGN | 19 | 37560 |
| MPGN | 27 | **54370** |
| COLLAGENOFIBROTIC GN (COLLAGEN TYPE 3 GLOMERULOPATHY) | 1 | 84087 |
| FIBRILLARY OR IMMUNOTACTOID GN | 3 | 91137 |
| FIBRONECTIN GLOMERULOPATHY | 2 | 84090 |
| **LIGHT CHAIN DEPOSITION DISEASE** | 1 | 93558 |
| **CAKUT Syndrome** | 94 |  |
| **ARNOLD CHIARI Syndrome** | 1 | 1136 |
| **ANTIPHOSPHOLIPID SYNDROME** | 6 | 536 |
| **SICKLE CELL ANEMIA** | 1 | 232 |
| **HAEMOPHILIA** | 2 | NA |
| HAEMOPHILIA A | 1 | 98878 |
| HAEMOPHILIA B | 1 | 98879 |
| **HEPATIC FIBROSIS** | 2 | 1454 |
| **GOODPASTURE Syndrome** | 8 | 375 |
| **GRANULOMATOSIS WITH POLYANGIITIS** | 3 | 900 |
| **JOUBERT Syndrome** | 2 | 475 |
| **CAROLI DISEASE** | 3 | 53035 |
| **LEBER HEREDITARY OPTIC NEUROPATHY** | 1 | 104 |
| **ACUTE INTERMITTENT PORPHYRIA** | 1 | 738 |
| **IMMUNE THROMBOTIC THROMBOCYTOPENIC PURPURA** | 4 | 3002 |
| **SJOGREN Syndrome** | 1 | 289390 |
| **IGG4-RELATED RETROPERITONEAL FIBROSIS** | 2 | 49041 |

RCAD: Renal cysts and diabetes syndrome. FSGS: focal segmental glomerulosclerosis. MGN: membranous glomerulonephritis. MPGN: membranoproliferative glomerulonephritis. NA: not available

**Table S2**. Baseline characteristics and renal function tests among the most frequent RDs subgroups of the studied population.

|  | Alport syndrome  (n=15) | Amyloidosis  (n=9) | Hyperossaluria (n=9) | Rapidly progressive GN (n=41) | Medullar cystic kidney disease (n=14) | CAKUT (n=94) | Goodpasture Syndrome (n=8) | GSFS (n=45) | MGN (n=19) | MPGN (n=27) |
| --- | --- | --- | --- | --- | --- | --- | --- | --- | --- | --- |
| **Recipient characteristics** |  |  |  |  |  |  |  |  |  |  |
| Age at transplant (yrs), mean (SD) | 44.2 (13.6) | 55.1  (8.7) | 41.9  (10.9) | 50.83  (14.3) | 45.5  (18.5) | 45.46 (13.5) | 52.4  (13.4) | 53.7 (12) | 56.2 (7.8) | 45.7 (12.2) |
| Gender Male, n (%) | 13  (86.7) | 3  (33.3) | 7  (77.7) | 18  (43.9) | 6  (42.8) | 61 (64.9) | 2  (25.0) | 32 (71.1) | 17 (89.5) | 13 (48.1) |
| Previous transplantation, n (%) | 5  (33.3) | 0  (0) | 4  (44.4) | 9  (21.9) | 1  (7.1) | 19 (20.2) | 1  (12.5) | 7  (15.5) | 3  (15.7) | 9  (33.3) |
| **Donor characteristics** |  |  |  |  |  |  |  |  |  |  |
| Age (yrs), mean (SD) | 49.9 (13.9) | 61.6  (8.2) | 47.0  (10.0) | 57.2  (14.2) | 51.1  (19) | 51.69 (13.4) | 59.4  (15.3) | 58.6 (12.5) | 61.9  (9) | 51.3 (12) |
| Gender male, n (%) | 8  (53.3) | 3  (33.3) | 4  (44.4) | 20  (48.8) | 6  (42.9) | 52 (55.3) | 4  (50.0) | 26 (57.7) | 8  (42.1) | 15 (55.5) |
| Deceased donor, n (%) | 13  (86.7) | 8  (88.9) | 8  (88.8) | 35  (85.4) | 10  (71.4) | 85 (90.4) | 7  (87.5) | 44 (97.8) | 17 (89.5) | 25 (92.6) |
| **At discharge** |  |  |  |  |  |  |  |  |  |  |
| Serum creatinine (mg/dL), mean (SD) | 1.4  (0.5) | 1.54  (0.6) | 1.59  (0.8) | 1.66  (0.8) | 2.16  (1.7) | 1.70 (0.5) | 1.64  (0.5) | 1.73 (0.6) | 2.21 (1.2) | 1.55 (0.5) |
| Proteinuria (g/d), mean (SD) | 0.43  (0.4) | 0.32  (0.4) | 1.53  (1.7) | 0.38  (0.3) | 0.36  (0.3) | 0.35 (0.3) | 0.32  (0.2) | 0.54 (0.7) | 0.81 (0.8) | 0.39 (0.3) |
| **At 1 year post-transplant** |  |  |  |  |  |  |  |  |  |  |
| Serum creatinine (mg/dL), mean (SD) | 1.46  (0.6) | 1.47  (0.6) | 1.74  (0.7) | 1.61  (0.9) | 2.02  (1.2) | 1.56 (0.5) | 1.49  (0.4) | 1.59 (0.6) | 1.97 (0.9) | 1.55 (0.5) |
| Proteinuria (g/d), mean (SD) | 0.26  (0.2) | 0.26  (0.1) | 0.45  (0.4) | 0.27  (0.2) | 0.34  (0.4) | 0.23 (0.2) | 0.28  (0.3) | 0.73 (1.4) | 1.08 (1.2) | 0.44 (0.8) |
| **At 2 years post-transplant** |  |  |  |  |  |  |  |  |  |  |
| Serum creatinine (mg/dL), mean (SD) | 1.42  (0.5) | 1.44  (0.6) | 1.53  (0.6) | 1.54  (0.6) | 2.03  (1.2) | 1.51 (0.5) | 1.37  (0.5) | 1.62 (0.7) | 1.81 (0.6) | 1.69 (0.7) |
| Proteinuria (g/d), mean (SD) | 0.28  (0.3) | 0.25  (0.2) | 0.5  (0.3) | 0.28  (0.4) | 0.54  (0.7) | 0.29 (0.5) | 0.25  (0.4) | 0.55 (1.1) | 1.33 (1.8) | 0.54  (1) |
| **At 5 years post-transplant** |  |  |  |  |  |  |  |  |  |  |
| Serum creatinine (mg/dL), mean (SD) | 1.59  (0.7) | 1.24  (0.2) | 1.72  (0.6) | 1.43  (0.5) | 1.8  (0.7) | 1.68 (0.7) | 1.52  (0.6) | 1.47 (0.56) | 2.31 (1.3) | 1.8 (0.9) |
| Proteinuria (g/d), mean (SD) | 0.47  (0.5) | 0.17  (0.1) | 0.29  (0.2) | 0.33  (0.5) | 0.36  (0.3) | 0.33 (0.5) | 0.4  (0.2) | 0.41 (0.7) | 1.02 (1.2) | 0.39 (0.5) |
| **Transplant failure, n (%)** | 1  (6.7) | 0  (0) | 1  (11.1) | 6  (14.6) | 4  (28.6) | 15  (15.9) | 0  (0) | 11 (24.4) | 6  (66.6) | 4  (14.8) |
| **Deceased with functioning kidney transplant, n (%)** | 1  (6.7) | 1  (11.1) | 1  (11.1) | 4  (9.8) | 0  (0) | 6  (6.4) | 0  (0) | 1  (2.2) | 2  (22.2) | 3  (11.1) |
